# Comparative analysis and integrative classification of NCI60 cell lines and primary tumors using gene expression profiling data

**DOI:** 10.1186/1471-2164-7-166

**Published:** 2006-07-03

**Authors:** Huixia Wang, Shuguang Huang, Jianyong Shou, Eric W Su, Jude E Onyia, Birong Liao, Shuyu Li

**Affiliations:** 1 Integrative Biology, Lilly Research Laboratories, Eli Lilly and Company, Lilly Corporate Center, Indianapolis, IN 46285, USA; 2 Global Discovery & Development Statistics, Lilly Research Laboratories, Eli Lilly and Company, Lilly Corporate Center, Indianapolis, IN 46285, USA; 3 Cancer Discovery Research, Lilly Research Laboratories, Eli Lilly and Company, Lilly Corporate Center, Indianapolis, IN 46285, USA; 4 Department of Statistics, North Carolina State University, 2501 Founders Drive, Raleigh NC 27695, USA

## Abstract

**Background:**

NCI60 cell lines are derived from cancers of 9 tissue origins and have been invaluable *in vitro *models for cancer research and anti-cancer drug screen. Although extensive studies have been carried out to assess the molecular features of NCI60 cell lines related to cancer and their sensitivities to more than 100,000 chemical compounds, it remains unclear if and how well these cell lines represent or model their tumor tissues of origin. Identification and confirmation of correct origins of NCI60 cell lines are critical to their usage as model systems and to translate *in vitro *studies into clinical potentials. Here we report a direct comparison between NCI60 cell lines and primary tumors by analyzing global gene expression profiles.

**Results:**

Comparative analysis suggested that 51 of 59 cell lines we analyzed represent their presumed tumors of origin. Taking advantage of available clinical information of primary tumor samples used to generate gene expression profiling data, we further classified those cell lines with the correct origins into different subtypes of cancer or different stages in cancer development. For example, 6 of 7 non-small cell lung cancer cell lines were classified as lung adenocarcinomas and all of them were classified into late stages in tumor progression.

**Conclusion:**

Taken together, we developed and applied a novel approach for systematic comparative analysis and integrative classification of NCI60 cell lines and primary tumors. Our results could provide guidance to the selection of appropriate cell lines for cancer research and pharmaceutical compound screenings. Moreover, this gene expression profile based approach can be generally applied to evaluate experimental model systems such as cell lines and animal models for human diseases.

## Background

Cell lines derived from primary tumor tissues have provided a valuable tool for the understanding of cancer biology at the molecular level. Ever since the development of Hela, the first human cancer continuous cell line by George Gey, Margaret Gey and Mary Kubicek more than 50 years ago, cancer cell lines have been instrumental as *in vitro *model systems in cancer research [1]. Much of the knowledge that we have today on fundamental processes in cancer cells has largely depended on the use of cell lines. In addition, since cancer cell lines provide an unlimited source of malignant cells, they are widely used in screening for anti-cancer drugs. However, because cells cultured *in vitro *lack the overall tissue architecture including tumor microenvironment, the value of cancer cell lines depends on if and to what extent cancer cell lines represent primary tumors that they are derived from. Some cancer cell lines undergo phenotypic and genotypic changes due to genomic instability. Many factors such as cross-contamination can cause cell line misclassification [2]. A recent study of 500 leukemia cell lines determined that 15% of these cell lines had been misclassified [3].

Several approaches have been utilized to authenticate cancer cell lines. The ability to form tumors when cell lines were transplanted subcutaneously into nude mice allows a direct comparison of histopathology between tumors formed in nude mice and the human tumors of origin [4]. Efforts have been made to delineate morphological features of cell lines in comparison with archival tumor tissues that the cell lines are derived from [5,6]. At molecular levels, expression of key proteins such as HER2/neu and p53 in breast and non-small cell lung cancer cell lines and their corresponding tumors have been assessed using immunohistochemistry [5,6]. Widely used in forensic analysis, DNA finger printing has been a valuable technique in analyzing loss-of-heterozygosity and microsatellite alterations [7]. Through current finger printing technology, one can not only confirm the identity of established cell lines and identify new cell lines, but also evaluate the purity of a cell culture [3].

The advent of high-throughput technologies, together with the completion of human genome sequencing project has created a new paradigm of understanding biology by simultaneous measurement of tens of thousands of genes in each biological sample. Numerous studies have employed microarray technology for classification and characterization of cancers. Comparisons between breast [8] or lung tumors [9] and tumor tissue derived cell lines have been attempted by gene expression profiling. However, a two-way hierarchical clustering generated dendrograms with cell lines and primary tumors falling into two isolated groups [8]. Recently, a tissue similarity index was developed to compare cell line and primary tumor gene expression profiling data using expression of tissue specific genes [10]. However, this approach that depends solely on tissue specific gene expression is problematic since genes not selected in the analysis may represent key pathways in cancer development.

NCI60 represents the most commonly used cancer cell lines in cancer research and drug screening. NCI60 cell lines have been extensively characterized by karyotyping [11], gene expression profiling [12,13] and protein arrays [14]. Their sensitivities to more than 100,000 compounds have been measured by the National Cancer Institute's Developmental Therapeutics Program (DTP) [15]. Here we report a direct comparison between NCI60 cell lines and 9 primary tumor types using publicly available gene expression profiling data generated from more than 500 primary tumor samples. We used Pearson's correlation coefficients to assess the similarities between cell lines and primary tumors. Correlation coefficients between a cell line and its presumed tumor of origin were tested against those between the cell line and other tumors to examine if the overall genome expression profiles derived from the cell line most closely resemble those derived from its presumed tumor origin. Furthermore, supervised learning methods were applied to classify cell lines into subtypes of cancer or into different tumor developmental stages for lung, CNS cancers and acute leukemia where clinical data of primary tumor samples were available. Our results suggest that the majority of the NCI60 cell lines are representative of their corresponding tumor types and thus provide suitable model systems for the study of cancer malignancies.

## Results

To obviate fundamental difference inherent in different array platforms, we focused our analysis on gene expression data from NCI60 cell lines and primary tumors (Table 1) based on Affymetrix U95Av2 oligonucleotide array platform. All the datasets represent the largest study of each cancer type to ensure statistical significance in our testing.

### Confirmation of tumor origins for NCI60 cell lines

**Figure 1:**
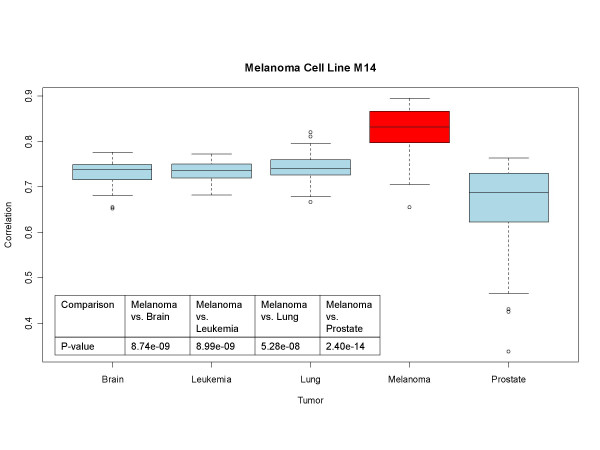
Melanoma cell line M14 represents its corresponding tumor type, as suggested by correlations of gene expression profiles between the cell line and primary tumors. The y-axis represents Pearson's correlation coefficients of gene expression profiles between the cell line and primary tumors. Labeled on x-axis are tumor types. Presumed tumor origin for the cell line is red-colored. The p-values of ANOVA tests for mean correlation coefficients are indicated in the table.

**Figure 2:**
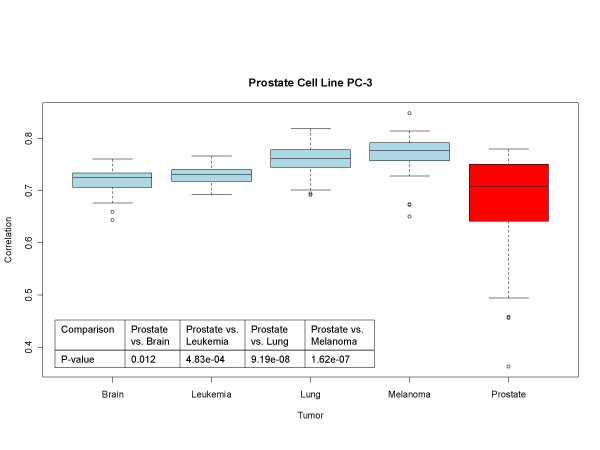
Prostate cell line PC-3 does not represent its corresponding tumor type, as suggested by correlations of gene expression profiles between the cell line and primary tumors. The y-axis represents Pearson's correlation coefficients of gene expression profiles between the cell line and primary tumors. Labeled on x-axis are tumor types. Presumed tumor origin for the cell line is red-colored. The p-values of ANOVA tests for mean correlation coefficients are indicated in the table.

We used Pearson's correlation coefficients of global gene expression profiles between cell lines and primary tumor samples to measure their similarities. We defined that a cell line is representative of its tumor origin if there is no other tumor type that has a gene expression profile with a significantly higher correlation to the cell line than the presumed tumor origin (p value < 0.05). Based on these criteria, the results of our analysis suggest that 51 of 59 NCI60 cell lines are representative of their corresponding tumors of origin (Table 2). All of colorectal cancer, leukemia, melanoma and ovarian cancer and most of breast cancer (6/7), renal carcinoma (7/8) and non-small cell lung cancer (7/9) cell lines have gene expression profiles most similar to the corresponding primary tumors. However, none of the 2 prostate cancer cell lines appears to represent their tumor origin based on our analysis. As illustrated in Figure 1, the mean of the correlation coefficients of gene expression between a melanoma cell line M14 and melanoma primary tumor samples is significantly higher than those between M14 and other tumor types. In contrast, the mean of the correlations between a prostate cell line PC-3 and prostate tumors is significantly smaller than that between PC-3 and melanomas or lung cancers (Figure 2), indicating that the overall gene expression profile of PC-3 is more similar to melanomas or lung cancers than to prostate cancers. Since there are outliers in the datasets as indicated in the box plots in Figure 1 and 2, we performed two non-parametric tests, the Wilcoxon test and the test for median difference, and obtained similar results (data not shown).

### Subclassification of lung cancer cell lines

**Figure 3:**
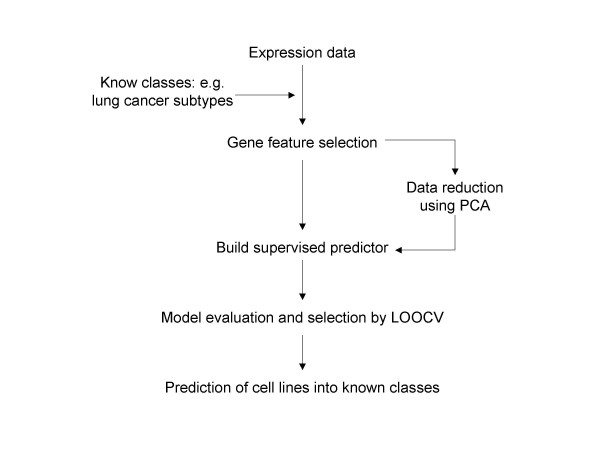
Classification of cancer cell lines using supervised learning methods. PCA: principal component analysis. LOOCV: leave-one-out cross validation.

Lung cancers are generally classified into two major types, non-small cell lung carcinoma (NSCLC) and small cell lung carcinoma (SCLC). Pulmonary carcinoid tumors are grouped with SCLCs because of their neuroendocrine features. NSCLC is further categorized into adenocarcinoma, squamous cell carcinoma and large cell carcinoma. The majority of lung carcinomas are heterogeneous and contain a mixture of different cell types. However, they are only classified as mixed carcinomas when the minority cell types exceed a threshold. Cell lines established from primary tumor biopsies may be misclassified because of heterogeneity in lung cancers. To investigate what subtypes that the lung cell lines most likely represent, we used supervised learning approaches (Figure 3) to predict the subtypes of the 7 non-small cell lung cancer cell lines that we had identified to be representative of primary lung tumors (Table 2).

We first selected gene features that best define the 4 individual groups of primary lung tumors: adenocarcinoma, squamous cell carcinoma, pulmonary carcinoid, and small cell carcinoma. Analysis of variance was carried out to identify genes differentially expressed between any two of the 4 subtypes. Six such pair-wise comparisons resulted in 359 probe sets that exhibited differential expression in 5 of 6 comparisons (p < 0.005). Data reduction using principle component analysis (PCA) and subsequent building of classification models were carried out as described in Material & Methods. The training dataset for supervised learning contains gene expression data from 186 primary lung cancer samples [16] that include 139 adenocarcinomas, 21 squamous cell lung carcinomas, 20 pulmonary carcinoids and 6 small cell lung cancers. Multiple models were built using different number of principle components, and we chose 4 principle components as the discriminants and LDA as the classification method that combined to minimize the error rate in leave-one-out cross validation (LOOCV). Detailed LOOCV results are provided in Table 4 [see Additional file 1]. Based on our classification model, 6 of the 7 cell lines we tested were classified as adenocarcinomas and NCI-H322M was classified into the squamous cell carcinoma subtype (Table 3).

**Figure 4:**
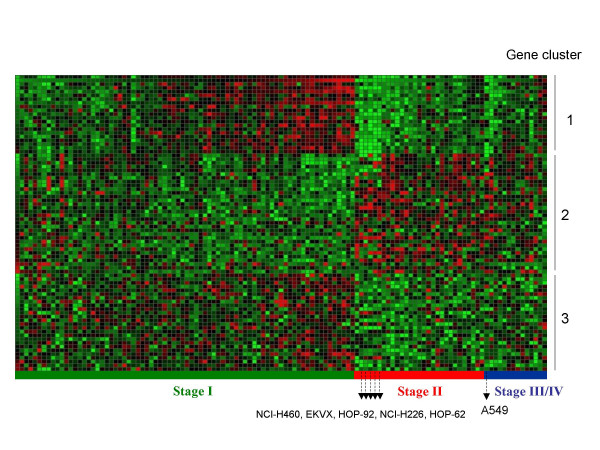
Subclassification of 6 lung adenocarcinoma cell lines into tumor stages. Gene expression of the 79 gene discriminant is illustrated by a heat map. Red and green represent high and low levels of expression respectively. The y-axis represents 79 genes and the x axis represents 113 lung adenocarcinomas and 6 NSCLC cell lines. 6 cell lines are labeled and 113 adenocarcinomas are grouped in to stage I, II and III/IV.

Other than the tissue of origin, cancers can also be classified by the stage depending on how far the cancer has spread and by the grade that describes how similar to normal cells that cancer cells appear under the microscope. In the primary lung cancer gene expression profiling dataset [16], cancer stage annotation was provided for 113 adenocarcinomas. Among them, 76, 24 and 13 patients are classified into stage I, stage II and stage III/IV, respectively. Using similar supervised learning methods (Figure 3), we attempted to classify the 6 cell lines that were identified to represent adenocarcinmas with respect to tumor stages. We chose 79 genes and kNN (k = 6) as the discriminant and the classification method, respectively, to build a model that has a minimal error rate in LOOCV (Table 5) [see Additional file 1]. Among the 6 adenocarcinoma cell lines, 5 were classified into the stage II group and A549 was classified into the stage III/IV group (Table 3). Gene expression profiles of the 79 gene feature, as illustrated by a heat map (Figure 4), demonstrates that they are expressed at similar levels between stage II and stage III/IV groups but exhibited a distinct expression pattern in stage I patients. For example, gene cluster 2 includes genes that are up-regulated in stage II and III/IV patients versus the stage I group. Over expression of many cluster 2 genes in stage II or later is not surprising as gene descriptions provided by Affymetrix probe set annotation [17] reveals some of these genes such as PTHLH, homeo box B7, a transcriptional activator that functions in angiogenesis, immediate early response 3, angiopoietin-like 2 are known to play a role in angiogenesis and others including several collagen family genes are involved in extracellular remodeling during tumor spread. Similar expression patterns of these marker genes observed in the 6 cell lines when compared with stage II and III/IV patients (Figure 4) strongly suggest that these cell lines can be used as ideal models for late stage lung adenocarcinomas.

### Subclassification of CNS cancer cell lines

Malignant gliomas are the most common type of brain tumors. Recent investigations have developed a gene expression profiling approach to delineate molecular features of gliomas and to classify high-grade gliomas including glioblastoma and anaplastic oligodendrogliomas [18]. Global gene expression data have been generated for 14 histologically classic glioblastomas and 7 anaplastic oligodendrogliomas [18]. In the NCI60 panel, all of the CNS cancer cell lines are derived from gliomas. To determine which high-grade gliomas each of the CNS cancer cell lines is most suitable as a model system for, we again used supervised learning algorithms to classify the 4 CNS cell lines representative of primary gliomas (Table 2). We tested the top 20 genes that are most significantly differentially expressed between glioblastomas and anaplastic oligodendrogliomas. In our final model, we used the top 3 genes and kNN (k = 3) classification algorithm that gave rise to an error rate of zero in LOOCV (Table 6) [see Additional file 1]. When this classification model was applied to the 4 CNS cell lines, all of them were classified as glioblastomas (Table 3).

### Subclassification of leukemia cell lines

Classification of acute leukemias is based on the observation of variable clinical outcome and difference in nuclear morphology. Traditionally, acute leukemias are classified into acute lymphoblastic leukemias (ALL) that arise from lymphoid precursors and acute myeloid leukemias (AML) that arise from myeloid precursors. 25% of ALL carrying a chromosomal translocation involving the mixed-lineage leukemia gene (MLL) have a particularly poor clinical outcome and are recently classified as a separate category MLL. Distinct gene expression profiles have been observed between ALL, AML and MLL in several studies of acute leukemias using gene expression profiling [19,20]. Here we took advantage of available clinical classifications of 72 leukemia samples that were confirmed by their gene expression profiling signature to classify the leukemia cell lines in the NCI60 panel. After testing of different number of genes that are most differentially expressed between the three subtypes and different classification methods, we used a 12 gene signature and the kNN (k = 7) algorithm to build our model (Table 7) [see Additional file 1]. Table 3 indicates that 5 of 6 cell lines are categorized into the AML group and CCRF-CEM was regarded as an ALL cell line.

## Discussion

Cancer cell lines have served as the primary experimental system for exploring cancer molecular biology and pharmacology. Although the value of cell lines in cancer research and anti-tumor compound screening is much appreciated, there is continued skepticism that cell lines under-represent the features of the primary tumors that they were derived from. Previous studies to some extent addressed these concerns by applying experimental approaches such as DNA finger printing to validate and authenticate cancer cell lines. In the last several years, microarray technology has been used to generate gene expression data for hundreds of tumor samples and provided a new paradigm of molecular based cancer classification. Previous work using gene expression profiling to compare cell lines and primary tumors has only focused on individual cancer types. A systematic effort in this area has been lacking to investigate if NCI60, the panel of cancer cell lines most widely used in cancer research and drug screening, represent their tumors of origin. Here we describe a novel approach to compare cell lines and primary tumors by computational analysis of publicly available gene expression profiling data of NCI60 cell lines and more than 500 primary tumor specimens. We were able to not only provide evidence to determine if a cancer cell line is correctly labeled to represent its corresponding tumor origin, but also classify a cell line into tumor subtypes or stages that the cell line may be most appropriate as a model system.

In contrast to some perceptions, our analysis suggested that most cancer cell lines are representative of their original tumor types. Global gene expression profiles in 51 of 59 NCI60 cell lines are most similar to that of their corresponding tumor origins. Although 8 cell lines have gene expression profiles more similar to tumor types other than their presumed origins, they do have strong correlations to their corresponding tumor types with correlation coefficients in the range of 0.7 (Table 2). Extensive experimental follow-up studies are necessary to clarify our computational analysis. However, there are several possible explanations for some of the discrepancies between the labels of these 8 cell lines and their gene expression profiles. Both prostate cell lines PC-3 and DU-145 had expression profiles with lower correlation coefficients to prostate cancers than other tumor types (Figure 2; data not shown). The lack of maximal correlation could be due to that PC-3 and DU-145 are androgen independent but most primary prostate cancers are androgen dependent [21,22]. The hormonal dependence affects cell growth and may cause significant changes of gene expression profiles [23]. Since the two cell lines were initiated from bone and brain metastases of prostate adenocarcinomas respectively [21,22], an alternative explanation is that the progenitor cells that they are derived from had lost the gene expression patterns of differentiated cells from the prostate. Among the 7 breast cell lines, only NCI/ADR-RES did not have an expression profile most similar to breast cancer biopsies. NCI/ADR-RES, originally known as MCF7/adr, is adriamycin resistant and was established through selection of MCF7 cells that are resistant to stepwise increasing concentrations of adriamycin [24]. Therefore, it is possible that adriamycin resistant MCF7 cells had altered gene expression profiles that are characteristic of cells from breast tissue. Indeed, it has been shown that in drug resistant cell lines, expression of some genes are induced during the selection process [24-26]. Upon further inspection of our correlation analysis results, we discovered that NCI/ADR-RES gene expression profile is most similar to those of ovarian cancers (data not shown). This finding is consistent with a recent report that NCI/ADR-RES is strikingly similar to an ovarian cancer cell line OVCAR8 based on a karyotyping study [11] and therefore supports our approach in using global gene expression profiles to evaluate the similarity between cell lines and primary cancer tissues.

Because we were able to obtain clinical annotations of the tumor samples used in gene expression profiling studies [16,18,19], 7 non-small cell lung cancer, 4 CNS cancer and 3 leukemia cell lines were classified into tumor subtypes or stages using supervised learning methods. 6 of the 7 NSCLC lines were classified as adenocarcinomas. This result has clarified the confusion in the literature regarding classification of some NSCLC cell lines. For example, NCI-H226 has been annotated as an adenocarcinoma cell line in some studies [27] but as a squamous carcinoma cell line in others [28]. Our supervised learning based classification using more than 100 primary tumor samples as the training dataset strongly suggest NCI-H226 is of adenocarcinoma origin. We also predicted tumor stages for the 6 adenocarcinoma cell lines. A549 falls into the stage III/IV group and the other 5 were predicted to represent stage II tumors (Table 3). Since the 79 gene feature that we used to build the classification model exhibited a similar expression profile between the stage II and stage III/IV patients (Figure 4), the 5 cell lines classified as stage II tumors could be in the stage III/IV group as well. Nevertheless, our results are consistent with the fact that cancer cell lines are generally derived from late stage cancers that have accumulated necessary genetic mutations for unlimited growth *in vitro*.

Our prediction of leukemia subtypes for 4 of the 6 acute leukemia lines is not in agreement with its description provided by American Type Culture Collection (ATCC). Specifically, SR and RPMI-8226 are classified into the AML category. However, they are lymphoma and myeloma cell lines, respectively, and therefore we recognize it would be inappropriate to attempt to classify them into either AML or ALL since lymphoma and myeloma are pathologically different than leukemia. Moreover, K-562 is also classified into the AML class, but it is derived from a patient with chronic myeloid leukemia (CML). Similarly, it would be inappropriate to classify it into one of the acute leukemia subtypes, AML or ALL. MOLT-4 is documented to be derived from a patient with ALL. However, we classified it into the myelogenous origin because it exhibits gene expression patterns more similar to AML than ALL (Table 3; data not shown). Characterization using immunological, cytogenetic and molecular biology approaches has clearly confirmed the identify of MOLT-4 as an ALL cell line [29]. One possible explanation for our misclassification is that we built classifiers based on gene expression profiles of 20 ALL and 28 AML patients from a single cohort. Therefore, even we achieved an error rate of zero in leave-one-out cross validation, it is possible that our classifier is not generally applicable. When microarray data on more ALL and AML patients from different cohorts become available in the future, we might be able to more accurately classify leukemia cell lines by building an improved classifier.

Attempts have been made in the past for comparison and integrative classification of primary tumors and tumor derived cell lines using gene expression profiling [8,9]. However, hierarchical clustering analysis generated dendrograms with cell lines and primary tumors falling into two distinct branches [8]. We also observed such separations when array datasets for NCI60 and 9 primary tumor types were subjected to hierarchical clustering (data not shown). One possible explanation is that gene expression differences between cell lines of different tumor origins are overshadowed by more significant differences between cell lines and primary tumors. Here we developed a simple analysis of correlation coefficients as a metric to measure the similarity between each cell line and different tumor types. In our approach, the general differences between cell lines and primary tissue samples would not interfere with comparisons between a cell line and multiple tumor types. Unlike previous studies that artificially selected genes differentially expressed between cell lines and primary tumors [9], our testing is robust and unbiased as we only removed genes that are deemed unexpressed in more than 80% of the array datasets. More significantly, to our knowledge, this is the first attempt to predict tumor subtypes or stages that the cell lines are suitable as model systems by applying supervised learning methods that have error rates close to zero in cross validations.

We also recognize the limitations in our study. First, gene expression profiles in cell culture *in vitro *may not reflect gene expressions evaluated when cells are grown *in vivo*. A recent study has shown that although two glioblastoma cell lines (U251 and U87) have disparate gene expression profiles when grown in monolayer cell cultures, they had similar gene expression patterns when grown as intracerebral xenografts in nude mice [30]. Therefore, a more insightful approach would be comparing gene expression profiles between primary human tumors and cell lines grown in xenograft models when such data become available. Second, correlation based analysis only provides hints on if a cell line is more representative of its presumed tumor origin than other tumor types, but does not provide a quantative measure on how close the cell line represents the corresponding cancer. Third, subclassification of cancer cell lines is not in fact available. Therefore, it is not possible to experimentally validate our prediction. Our classification based on supervised learning approach only provides suggestions on which cancer subtype that a cell line most closely represents. Fourth, overall genomic gene expression profiles were used in our statistical testing of gene expression correlations between cell lines and tumor samples. However, specific genes and pathways may be involved in development of different types of cancer. When this manuscript was in preparation, a study published by Minn et al. has identified 54 genes differentially expressed between one of the NCI60 breast cell lines, MDA-MB-231 and its single-cell-derived sub-line that has significantly higher lung metastatic activity [31]. Expressions of this lung metastasis signature not only mediate lung metastasis of MDA-MB-231 cells in mice, but are also significantly associated with breast cancer metastasis to lung in human [31], suggesting that the same set of genes plays a critical role in lung metastasis both in cell line models and in humans. Therefore, future analysis using expression of selected genes in known cancer development pathways would certainly shed light on the activity of these pathways in cancer cell lines in comparison with human tumors.

## Conclusion

We developed and applied a novel approach for systematic comparative analysis and integrative classification of NCI60 cell lines and primary tumors. Comparative analysis suggested that 51 of 59 NCI60 cell lines we analyzed represent their presumed tumors of origin. Some of the 51 cell lines with the correct origins were further classified into different subtypes of cancer or different stages in cancer development based on supervised learning methods. Our results could provide guidance to the selection of appropriate cell lines for cancer research and pharmaceutical compound screenings. Furthermore, this gene expression profile based approach can be generally applied to evaluate experimental model systems such as cell lines and animal models for human diseases.

## Methods

### Data source

Gene expression profiling data on NCI60 cell lines provided by NCI's DTP program [32] are based on Affymetrix U95Av2 oligonucleotide array platforms. While oligonucleotide arrays measure the amount of mRNA in a single sample, gene expression data generated using cDNA array platforms are ratios of expression values in experimental samples over those in a reference sample. The fundamental difference between the two array platforms poses a technical barrier in integrative analysis of gene expression data based on these two different platforms. In addition, probe sets representing the same genes are designed differently on different Affymetrix oligonucleotide arrays. These probe sets may behave differently in array hybridization and produce discordant expression values. Therefore, we chose only U95Av2 oligonucleotide array based data in publicly available gene expression profiling datasets on primary tumors (Table 1).

Gene expression data on NCI60 cell lines and primary tumor samples were downloaded from URL addresses shown in Table 1. All datasets were generated with Affymetrix U95Av2 arrays. Gene expression data of lung [16], prostate [33], CNS [18] cancers and leukemia [19] were originally generated with Affymetrix MAS4 software. We downloaded the .cel files and produced more accurate gene expression data using Affymetrix MAS5 algorithm with trimmed mean values normalized to 500. A trimmed mean is the average value after removing the lowest 2% and the highest 2% of all expression values. The downloaded array data for NCI60 cell lines and melanomas were in MAS5 format and we re-normalized the data by setting the trimmed means to 500. Data are only available for 59 of the NCI60 cell lines. For breast, colon, ovary and kidney cancers, we were only able to obtain MAS4 gene expression data and similarly, these data were normalized with trimmed means equal to 500.

### Pre-processing

We compiled the gene expression data for a total of 506 samples, after averaging the expression values over the technical replicates in the lung dataset and in NCI60 cell lines. The Affymetrix MAS5 algorithm provided the "Absent/Present" calls for each probe set to indicate the expression level is below/above the threshold of detection. An "Absent" call means the hybridization signal derived from the perfect match probe is not provably different than that derived from the mismatch probe. In MAS4 formatted datasets that we downloaded, "Absent/Present" calls are not provided and we arbitrarily assigned the "Absent" calls to the probe sets with gene expression values below 40. We chose 40 as a threshold to make the "Absent/Present" calls so that the percentage of probe sets with "Present" calls are similarly to that in the MAS5 datasets. To correct the systematic bias, we applied quantile normalization across samples following the method in Bolstad et al. [34] to the compiled dataset. We then filtered out the probe sets that received "Absent" calls in more than 80% of the samples as well as the Affymetrix control probe sets, and this left 11,482 probe sets for further analysis. We further performed a log2 transformation, and then standardized each sample to a mean of 0 and standard deviation of 1.

### Comparative analysis of cell lines and primary tumors

We used Pearson's correlation coefficients to assess the similarities between cell lines and the primary tumors. Since different algorithms implemented in Affymetrix MAS4 and MAS5 affect overall gene expression profiles, we separated primary tumor samples into two groups that include array data in MAS4 and MAS5 format, respectively (see Table 1). We computed the Pearson's correlation coefficients of gene expression profiles between each of the 59 NCI60 cell lines and each primary tumor sample. For each cell line, we performed pairwise t-test with Scheffe multiple comparison adjustment comparing the mean of the correlation coefficients between the cell line and its corresponding tumor to the means of those between the cell line and the other tumor types in the same data format group, successively. We defined that a cell line is representative of its tumor origin if there is no other tumor type that has a significantly higher correlation of gene expression profiles with the cell line than the presumed tumor origin at a significance level of 0.05.

### Integrative classification

#### Feature selection

Since many genes exhibit near constant expression levels across the tumor samples, we first carried out a feature selection for each classification to find a minimum set of features that are useful for classification. For the classification of the lung cancer subtypes, we identified the 359 genes that are differentially expressed between at least 5 pairs of subtypes with a raw p-value cutoff of 0.005. Then we performed principle component analysis on those 359 genes and chose the first *p *principle components. For the classification of tumor stages of lung adenocarcinoma, subtypes of CNS and subtypes of leukemia, we chose the top *p *most significant genes that are differentially expressed across classes, with an attempt to balance the number of up-regulated genes, the number of down-regulated genes and the total number of genes differing between each pair of the classes. The criteria for the selection of *p *are discussed in the following "classification methods" section.

#### Classification method

We compared different classification methods and found that two simple classifiers, linear discriminant analysis (LDA) and k-nearest-neighbor (kNN) performed reasonably well. The *k *in kNN is chosen between 3–7 so that the leave-one-out cross-validation (LOOCV) error rate is the smallest. Leave-one-out cross-validation involves removing one data object in turn from the training set, training a classifier on the remaining objects and then testing on the removed one. The proportion of errors counted throughout this process is called leave-one-out cross-validation error rate. The classifiers were built on the features with *p *genes or *p *principle components. The value *p *was chosen to be the smallest one that satisfies two criteria: (1) the leave-one-out cross validation error rate of the classifier is smaller than 0.01; and (2) the 3 consecutive classifiers built on the features with *p*, *p+1 *and *p+2 *genes or principle components give consistent predictions. The classifier for each classification problem was chosen between LDA and kNN by comparing the LOOCV error rates.

## Authors' contributions

HW and SL carried out data analysis. SH, BL and SL designed study. SH, JS, EWS, JEO, BL and SL interpreted results. HW and SL drafted the manuscript. SH, JS, EWS, JEO and BL revised the manuscript.

## Supplementary Material

Additional File 1 Table 4, Table 5, Table 6, Table 7. Click here for file

## Figures and Tables

**Table 1:** Gene expression profiling datasets on NCI60 cell lines and primary tumors analyzed in this study.

Reference	Cancer Type	Sample Size	Data Format	URL for Data Downloading
[13]	NCI60 cell lines	-	MAS5	
[16]	Lung	186	MAS5	
[33]	Prostate	52	MAS5	
[19]	Leukemia	72	MAS5	
[18]	CNS	21	MAS5	
[35]	Melanoma	29	MAS5	
[36]	Breast	26	MAS4	
[36]	Colon	23	MAS4	
[36]	Kidney	11	MAS4	
[36]	Ovary	27	MAS4	

**Table 2:** 51 of 59 NCI60 cell lines represent their presumed tumors of origin.

Tumor type	Cell lines representing correct tumor origins	Cell lines with incorrect tumor origins
Breast	MCF7, MDA-MB-435, BT-549, T-47D, MDA-MB-231, HS578T	NCI/ADR-RES
CNS	SNB-19, SNB-75, U251, SF-268	SF-295, SF-539
Colon	HT-29, COLO205, HCT-15, KM12, HCC-2998, HCT-116, SW-620	-
Kidney	A498, CAKI-1, RXF-393, 786-0, ACHN, TK-10, UO-31	SN12C
Leukemia	RPMI-8226, SR, CCRF-CEM, K-562, MOLT-4, HL-60	-
Lung	NCI-H226, NCI-H322M, NCI-H460, HOP-62, HOP-92, A549, EKVX	NCI-H23, NCI-H522
Melanoma	LOX IMVI, M14, MALME-3M, UACC-62, UACC-257, SK-MEL-2, SK-MEL-5, SK-MEL-28	-
Ovary	OVCAR-3, IGROV1, SK-OV-3, OVCAR-4, OVCAR-5, OVCAR-8	-
Prostate	-	PC3, DU-145

**Table 3:** Classification of non-small cell lung cancer, CNS cancer and leukemia cell lines into tumor subtypes or tumor stages.

Cell lines	No. of cell lines	Known classes	Gene feature	Classification method	LOOCV error rate	Prediction results
Lung	7	AD, SQ, COID, SCLC	4 PC	LDA	0.01	AD (NCI-H226, NCI-H460, HOP-62, HOP-92, A549, EKVX) SQ (NCI-H322M)
Lung AD	6	stage I, stage II, stage III/IV	79 genes	kNN (k = 6)	0	stage II (NCI-H226, HOP-62, HOP-92, EKVX, NCI-H460) stage III/IV (A549)
CNS	4	CG, CO	3 genes	kNN (k = 3)	0	CG (SNB-19, SNB-75, U251, SF-268)
Leukemia	6	ALL, AML, MLL	12 genes	kNN (k = 7)	0	AML (RPMI-8226, SR, K-562, MOLT-4, HL-60) ALL (CCRF-CEM)

*AD: adenocarcinoma. SQ: squamous cell carcinoma. COID: pulmonary carcinoid tumor. SCLC: small cell lung cancer. CG: classic glioblastoma. CO: classic oligodendroglioma. ALL: acute lymphoblastic leukemia. AML: acute myeloid leukemia. MLL: mixed lineage leukemia. LDA: linear discriminant analysis. kNN: k nearest neighbour. PC: principal components. LOOCV: leave-one-out cross validation.
