# Supplementary material for: Comparative analysis and integrative classification of NCI60 cell lines and primary tumors using gene expression profiling data
Source: BMC Genomics. 2006 Jul 3;7:166. doi: 10.1186/1471-2164-7-166 (PMC1525183; doi:10.1186/1471-2164-7-166)
Supplement: Additional File 1 — Table 4, Table 5, Table 6, Table 7. [file 1471-2164-7-166-S1.doc]

Table 4. Error rates of leave-one-out cross validation in classification of lung cancer subtypes using 186 primary tumor samples as the training dataset. Principle components (PCs) were generated from 359 probe sets that show differential expression between the 4 lung cancer subtypes. Classification models were built using two methods, linear discriminant analysis (LDA) and k-nearest neighbors (kNN).

| No. PCs | LDA | kNN (k=6) |  | No. PCs | LDA | kNN (k=6) |
| --- | --- | --- | --- | --- | --- | --- |
| 1 | 0.338 | 0.279 |  | 36 | 0.026 | 0.043 |
| 2 | 0.134 | 0.161 |  | 37 | 0.026 | 0.043 |
| 3 | 0.118 | 0.166 |  | 38 | 0.026 | 0.043 |
| 4 | 0.011 | 0.038 |  | 39 | 0.026 | 0.043 |
| 5 | 0.026 | 0.043 |  | 40 | 0.026 | 0.038 |
| 6 | 0.021 | 0.048 |  | 41 | 0.026 | 0.043 |
| 7 | 0.026 | 0.043 |  | 42 | 0.026 | 0.043 |
| 8 | 0.026 | 0.048 |  | 43 | 0.031 | 0.043 |
| 9 | 0.027 | 0.048 |  | 44 | 0.031 | 0.043 |
| 10 | 0.032 | 0.048 |  | 45 | 0.032 | 0.043 |
| 11 | 0.037 | 0.043 |  | 46 | 0.032 | 0.038 |
| 12 | 0.032 | 0.048 |  | 47 | 0.032 | 0.038 |
| 13 | 0.032 | 0.038 |  | 48 | 0.032 | 0.038 |
| 14 | 0.032 | 0.032 |  | 49 | 0.032 | 0.038 |
| 15 | 0.032 | 0.043 |  | 50 | 0.031 | 0.027 |
| 16 | 0.031 | 0.054 |  | 51 | 0.031 | 0.032 |
| 17 | 0.031 | 0.054 |  | 52 | 0.031 | 0.032 |
| 18 | 0.037 | 0.065 |  | 53 | 0.031 | 0.032 |
| 19 | 0.026 | 0.054 |  | 54 | 0.031 | 0.038 |
| 20 | 0.026 | 0.065 |  | 55 | 0.031 | 0.032 |
| 21 | 0.026 | 0.065 |  | 56 | 0.031 | 0.032 |
| 22 | 0.026 | 0.059 |  | 57 | 0.026 | 0.038 |
| 23 | 0.032 | 0.059 |  | 58 | 0.026 | 0.032 |
| 24 | 0.031 | 0.054 |  | 59 | 0.026 | 0.038 |
| 25 | 0.037 | 0.054 |  | 60 | 0.026 | 0.038 |
| 26 | 0.037 | 0.059 |  | 61 | 0.021 | 0.038 |
| 27 | 0.037 | 0.070 |  | 62 | 0.021 | 0.043 |
| 28 | 0.037 | 0.065 |  | 63 | 0.021 | 0.032 |
| 29 | 0.026 | 0.054 |  | 64 | 0.026 | 0.032 |
| 30 | 0.026 | 0.054 |  | 65 | 0.026 | 0.027 |
| 31 | 0.026 | 0.038 |  | 66 | 0.026 | 0.032 |
| 32 | 0.026 | 0.043 |  | 67 | 0.026 | 0.027 |
| 33 | 0.026 | 0.038 |  | 68 | 0.026 | 0.027 |
| 34 | 0.026 | 0.038 |  | 69 | 0.016 | 0.022 |
| 35 | 0.026 | 0.043 |  | 70 | 0.016 | 0.016 |

Table 5. Error rates of leave-one-out cross validation in classification of lung cancer stages using 113 lung adenocarcinoma samples as the training dataset. Gene features were selected that show most significant differential expression between the three groups, stage I, stage II, stage III/IV. Classification models were built using two methods, linear discriminant analysis (LDA) and k-nearest neighbors (kNN).

| No. Genes | LDA | kNN (k=6) | No. Genes | LDA | kNN (k=6) | No. Genes | LDA | kNN (k=6) |
| --- | --- | --- | --- | --- | --- | --- | --- | --- |
| 10 | 0.274 | 0.327 | 40 | 0.292 | 0.177 | 70 | 0.319 | 0.027 |
| 11 | 0.265 | 0.274 | 41 | 0.292 | 0.212 | 71 | 0.327 | 0.035 |
| 12 | 0.274 | 0.265 | 42 | 0.292 | 0.204 | 72 | 0.292 | 0.027 |
| 13 | 0.274 | 0.301 | 43 | 0.257 | 0.177 | 73 | 0.310 | 0.027 |
| 14 | 0.265 | 0.301 | 44 | 0.257 | 0.168 | 74 | 0.310 | 0.035 |
| 15 | 0.265 | 0.292 | 45 | 0.248 | 0.159 | 75 | 0.319 | 0.027 |
| 16 | 0.292 | 0.248 | 46 | 0.283 | 0.115 | 76 | 0.274 | 0 |
| 17 | 0.319 | 0.239 | 47 | 0.265 | 0.142 | 77 | 0.319 | 0 |
| 18 | 0.319 | 0.265 | 48 | 0.274 | 0.177 | 78 | 0.310 | 0 |
| 19 | 0.274 | 0.274 | 49 | 0.283 | 0.168 | 79 | 0.327 | 0 |
| 20 | 0.283 | 0.257 | 50 | 0.274 | 0.168 | 80 | 0.274 | 0 |
| 21 | 0.283 | 0.274 | 51 | 0.265 | 0.133 | 81 | 0.257 | 0 |
| 22 | 0.265 | 0.292 | 52 | 0.274 | 0.150 | 82 | 0.265 | 0 |
| 23 | 0.283 | 0.292 | 53 | 0.283 | 0.159 | 83 | 0.265 | 0 |

Table 6. Error rates of leave-one-out cross validation in classification of CNS cancer subtype using 21 primary tumor samples as the training dataset. Gene features were selected that show most significant differential expression between the two subtypes (glioblastoma and anaplastic oligodendroglioma). Classification models were built using two methods, linear discriminant analysis (LDA) and k-nearest neighbors (kNN).

| No. Genes | LDA | kNN (k=3) |
| --- | --- | --- |
| 1 | 0.095 | 0.095 |
| 2 | 0.048 | 0.048 |
| 3 | 0.095 | 0 |
| 4 | 0.095 | 0.048 |
| 5 | 0.095 | 0.048 |
| 6 | 0.143 | 0.048 |
| 7 | 0.048 | 0 |
| 8 | 0.048 | 0 |
| 9 | 0.095 | 0 |
| 10 | 0.095 | 0 |
| 11 | 0.095 | 0 |
| 12 | 0.095 | 0 |
| 13 | 0.095 | 0 |
| 14 | 0.143 | 0 |
| 15 | 0.048 | 0 |
| 16 | 0.286 | 0 |
| 17 | 0.095 | 0 |
| 18 | 0.238 | 0 |
| 19 | 0.333 | 0 |
| 20 | 0.143 | 0 |

Table 7. Error rates of leave-one-out cross validation in classification of leukemia subtype using 72 primary tumor samples as the training dataset. Gene features were selected that show most significant differential expression between the three subtypes (AML, ALL, MLL). Classification models were built using two methods, linear discriminant analysis (LDA) and k-nearest neighbors (kNN).

| No. Genes | LDA | kNN (k=7) |
| --- | --- | --- |
| 1 | 0.167 | 0.167 |
| 2 | 0.014 | 0.014 |
| 3 | 0.028 | 0.028 |
| 4 | 0.014 | 0.014 |
| 5 | 0.014 | 0.014 |
| 6 | 0.014 | 0.014 |
| 7 | 0 | 0 |
| 8 | 0.028 | 0 |
| 9 | 0.014 | 0 |
| 10 | 0 | 0 |
| 11 | 0 | 0 |
| 12 | 0 | 0 |
| 13 | 0.014 | 0 |
| 14 | 0.028 | 0 |
| 15 | 0.028 | 0 |
| 16 | 0.028 | 0 |
| 17 | 0.028 | 0 |
| 18 | 0.042 | 0 |
| 19 | 0.042 | 0 |
| 20 | 0.042 | 0 |
